# Computational Representation of White Matter Fiber Orientations

**DOI:** 10.1155/2013/232143

**Published:** 2013-08-20

**Authors:** Adelino R. Ferreira da Silva

**Affiliations:** Departamento de Engenharia Electrotécnica, Faculdade de Ciências e Tecnologia (FCT), Universidade Nova de Lisboa, 2829-516 Caparica, Portugal

## Abstract

We present a new methodology based on directional data clustering to represent white matter fiber orientations in magnetic resonance analyses for high angular resolution diffusion imaging. A probabilistic methodology is proposed for estimating intravoxel principal fiber directions, based on clustering directional data arising from orientation distribution function (ODF) profiles. ODF reconstructions are used to estimate intravoxel fiber directions using mixtures of von Mises-Fisher distributions. The method focuses on clustering data on the unit sphere, where complexity arises from representing ODF profiles as directional data. The proposed method is validated on synthetic simulations, as well as on a real data experiment. Based on experiments, we show that by clustering profile data using mixtures of von Mises-Fisher distributions it is possible to estimate multiple fiber configurations in a more robust manner than currently used approaches, without recourse to regularization or sharpening procedures. The method holds promise to support robust tractographic methodologies and to build realistic models of white matter tracts in the human brain.

## 1. Introduction

Diffusion magnetic resonance imaging (MRI) is an MRI method that is able to characterize the diffusion displacement of water molecules in structured tissues of the human brain [1]. The key idea behind diffusion MRI is that of anisotropic diffusion. In structured tissues water mobility is not always the same in all directions. Molecular motion is favored in directions aligned with bundles of parallel fibers, such as in the human brain's white matter. The natural diffusion of water molecules can reveal *in vivo* microscopic details about the architecture of both normal and diseased tissues. White matter fiber tractography is commonly implemented using the principal diffusion direction of the diffusion tensor imaging (DTI) model [2]. Popular fiber tracking approaches, such as the streamline tracking algorithm [3], uses the DTI model to extract the orientation dependence of the diffusion probability density function (PDF) of water molecules. However, the standard single-tensor DTI model is based on a Gaussian diffusion assumption; thus unable to resolve crossing and splitting of fiber bundles. Extended tensor models for fiber tracking based on mixture of Gaussian densities [4] and multitensor models [5] have been proposed to enable detection of multiple orientation distribution function (ODF) maxima per voxel.

On the other hand, several studies have shown that fiber tracking based on high angular resolution diffusion imaging (HARDI) techniques is improved and less sensitive to noise errors compared to tensor based tracking [6–8]. The application of these methods is based on the assumption that the principal directions extracted from the ODF can be interpreted as principal directions of the underlying fiber architecture. The most commonly used approach for identifying fiber directions is to extract the local maxima of the reconstructed ODF, where this function surpasses a certain threshold. Thresholding avoids selecting smaller ODF peaks that may appear due to noise. Typically, local maxima of the reconstructed ODF are located simply by selecting a large number of randomly sampled points on the sphere and searching within a fixed radius neighborhood [6]. For two-fiber populations, the major fiber is identified by the largest local maximum (the global maximum), and the minor fiber is identified by the second largest local maximum [9]. Some more sophisticated heuristics built on this basic approach have been proposed. For instance, in [10], a Quasi-Newton method is used to refine the position of each local maximum. Afterwards, duplicate local maxima and any insignificant spikes with function values smaller than some threshold are removed. In [11] a spherical Newton method was proposed. However, as shown in [12, 13], the peaks of the ODF profiles identified by these methods do not necessarily match the orientations of the distinct fiber populations. An approach using a mixture of von Mises-Fisher (vMF) distributions has been proposed in [14]. However, in [14] the model needs to fit a mixture of four vMF distributions by a nonlinear least-squares technique to synthesize diffusion ODF profiles, before fiber directions are estimated by local maxima algorithms.

All these procedures have in common the fact that they apply deterministic procedures to ODF reconstructed profiles in order to evaluate intravoxel principal fiber directions. In this paper we will refer to this class of deterministic procedures as the standard (Std) approach for intravoxel principal fiber mapping. The main purpose of the Std approach is to support tractographic methodologies, able to build realistic models of white matter tracts in the human brain. Probabilistic tractography algorithms have been developed to resolve fiber crossings at the intravoxel level under looser constraints than deterministic tracking methods. In particular, the approach of using the Std method for mapping intravoxel fiber directions in probabilistic tractography is pervasive in currently available packages, such as “MRtrix” (*find_SH_peaks*) [15], “Camino” (*sfpeaks*) [16], “DSI Studio” (*find_peak*) [9], and “FSL” (*peakfinder*) [17]. However, using deterministic intravoxel procedures to support probabilistic fiber mapping in the brain jeopardizes rigorous fiber tractography and may originate deficient maps of white matter fiber networks.

In this paper, we present a new methodology based on directional data clustering to represent white matter fiber orientations in magnetic resonance analyses for high angular resolution diffusion imaging. The method focuses on clustering data on the unit sphere, where complexity arises from representing ODF profiles as directional data. A clustered mixture-model approach to model directional ODF data based on von Mises-Fisher (vMF) distributions is used, in order to support the probabilistic estimation of intravoxel fiber directions. In this “clustered vMF” approach, each estimated voxel fiber direction is associated with a component of the fitted mixture of vMF distributions. Hence, each voxel fiber principal direction may be specified by the summary statistics of the estimated vMF component in the mixture.

Based on voxel ODF reconstructions, our method estimates intravoxel fiber directions by clustering mixtures of von Mises-Fisher distributions fitted to probabilistic distributions. As opposed to other approaches where mixture of vMF distributions are used to represent diffusion [14, 18], our method works directly with the sampled ODF distributions. It should be noted that the proposed clustered vMF method is not used for ODF reconstruction. The ultimate objective of our analysis is to estimate principal fiber directions using statistical clustering approaches, in order to support robust probabilistic tractographic algorithms [19]. Before applying the clustered vMF approach we need to obtain the ODF profiles at each voxel. For this purpose, in this paper we use the Generalized *q*-Sampling Imaging (GQI) method proposed in [9]. Similarly to other methods for ODF reconstruction, GQI uses shell or grid sampling schemes to extract information about the extent of diffusion anisotropy, and map vector fields that represent the fiber orientations at each voxel. The paper reports on the use of a GQI approach for reconstruction, but other methods could be used as well. For instance, the author's package “gdimap” [20] (see Section 3.5) is a free, open source software package that supports three different ODF reconstruction methods. Nevertheless, it should be emphasized that the aim of this work is to present a new methodology for fiber directional mapping and not to compare ODF reconstruction methods.

The rest of the paper is organized as follows. In Section 2 we introduce the theoretical basis of the computational methods that have been used to drive the experiments reported in Section 3. Two basic methodologies are analyzed: (i) the GQI method for ODF reconstruction, and (ii) the mixture of vMF distributions for fiber crossing mapping and directional estimation. These methodologies have been applied to simulated experiments as well as to real data experiments. In Section 3.1, we report on analyses for synthetic data simulation of crossing fibers. In Section 3.2 we compare the proposed clustered vMF approach for ODF orientation estimation with the Std approach for crossing fibers mapping via local maxima extraction.  Section 3.3, provides details on simulations with curved fiber bundle simulations. In Section 3.4 a publicly available DICOM data set from the “Advanced Biomedical MRI Lab, National Taiwan University Hospital” is analyzed. Section 3.5 provides details on the implementation environment used to support reproducible research. Finally, Section 4 draws conclusions and presents guidelines for future research.

## 2. Materials and Methods

In this work, the experiments, analyses, and implementation reported in Section 3 were performed using the GQI method for ODF reconstruction. In Section 2.1, we present a brief overview of the GQI method. The reconstructed ODF profiles are then used to estimate the orientation of voxel ODF profiles, using directional clustering based on mixtures of von Mises-Fisher (vMF) distributions. In Section 2.2, we present this new proposed approach for mapping fiber orientations.

### 2.1. GQI

The generalized *q*-sampling imaging method as proposed in [9] derives a Fourier transform relation between *k*-space and *q*-space imaging to estimate the ODF directly from diffusion MR signals. The GQI method is based on a relationship between the spin density function *Q*(**R**) and the diffusion-weighted signal *W*(**q**), through the cosine transform relation

(1) $$\begin{array}{c} Q\left(R\right)=\int W\left(q\right)\cos\left(2\pi q\cdot R\right)dq, \end{array}$$

where **R** is the diffusion displacement during the diffusion time Δ, and **q** is the coordinate in *q*-space. The diffusion-weighted signal *W*(**q**) can be estimated directly from any unbiased *q*-space sampling scheme, such as grid, shell, or nongrid sampling scheme [21]. In GQI, the measured spin density function *Q*(**R**) is used to derive the spin distribution function (SDF) *ψ*(**u**), which is assumed effectively as the ODF of interest. While the diffusion ODF is a probability distribution of the diffusion displacement, the SDF represents a quantitative distribution of the spins undergoing diffusion. The quantity of spins that undergo diffusion in a particular radial direction **u** is summarized by the spin density function

(2) $$\begin{array}{c} \psi \left(u\right)=\int_{0}^{L_{\Delta }}Q\left(Lu\right)dL, \end{array}$$

where *L*_Δ_ is the diffusion sampling length (Einstein length) within the diffusion time Δ. The relation between the acquired diffusion images *W*(**q**) and the spin density function *ψ*(**u**) is obtained from (1) and (2) to get

(3) $$\begin{array}{c} \psi \left(u\right)=L_{\Delta }\int W\left(q\right)\text{sinc}\left(2\pi L_{\Delta }q\cdot u\right)dq, \end{array}$$

where sinc(*x*) = sin(*x*)/*x* for all *x* except 0, and sinc(0) = 1. For computational purposes, the GQI reconstruction procedure is obtained from (3) by expressing *ψ*(**u**) as a weighted sum of sinc functions in the form

(4) $$\begin{array}{c} \psi_{s}\left(u\right)=L_{\Delta }\sum_{q=q_{1}}^{q_{m}}W\left(q\right)\text{sinc}\left(2\pi L_{\Delta }q\cdot u\right), \end{array}$$

where *m* is the total number of sampling points over *q*-space.

In summary, as shown in [9, 21], the SDF along each radial direction is equivalent to the ODF, and the ODF reconstruction procedure can be obtained by applying (4) to the sampled *q*-space data.

### 2.2. Fiber Mapping Based on Directional Data Clustering

The second main feature of the proposed methodology is concerned with multiple directional mapping. Starting with the raw HARDI signal acquired on a grid of *q*-space, the ODF profile is estimated at each voxel, considering a sampling density of unit vectors on a unit *𝕊* ^2^ grid. When a threshold is applied to the estimated ODF at each voxel, the nonthresholded unit vectors provide directional statistics information about the estimated ODF profile. The main ODF orientations at each voxel relevant for fiber tracking may be estimated by clustering the nonthresholded unit vectors. This directional clustering procedure has several advantages compared to traditional approaches for orientation mapping. In fact, current best practices perform multiple maxima extraction based on procedures which are very sensitive to the local modes that appear in the reconstructed ODFs. Signal noise and low sampling resolution yield deformed ODF reconstruction profiles, thus affecting accuracy and precision in multiple orientation evaluations. In contrast, estimating orientations from clustered directional data is much less sensitive to local modes in the reconstructed ODF profile. Moreover, the procedure is more robust to noise since it estimates orientations statistically from sampled data.

For directional clustering estimation, we consider a mixture of *k* von Mises-Fisher (vMF) distributions [22] that serves as a model for directional ODF profile data, corresponding to multiple fiber orientations. A mixture of *k* vMF distributions has a density given by

(5) $$\begin{array}{c} f\left(x\;|\;\Theta \right)=\sum_{h=1}^{k}\alpha_{h}f_{h}\left(x\;|\;\theta_{h}\right), \end{array}$$

where *f*_*h*_(**x** | ***θ***_*h*_) denotes a vMF distribution with parameter ***θ***_*h*_ = (*μ*_*h*_, *κ*_*h*_) for 1 ≤ *h* ≤ *k*, Θ = {*α*_1_,…, *α*_*k*_, ***θ***_1_,…, ***θ***_*k*_}, and the *α*_*h*_ are nonnegative and sum to 1. A *d*-dimensional unit random vector **x** ∈ *𝕊* ^*d*−1^ is said to have *d*-variate vMF distribution if its probability density function is given by

(6) $$\begin{array}{c} f_{h}\left(x\;|\;\mu ,\kappa \right)=c_{d}\left(\kappa \right)e^{\kappa \mu^{T}x}, \end{array}$$

where ||*μ*|| = 1, *κ* ≥ 0, *d* ≥ 2, and *c*_*d*_(*κ*) is a normalizing constant [23]. The density *f*_*h*_(**x** | *μ*, *κ*) is parameterized by the mean direction *μ* and the concentration parameter *κ*. The *κ* parameter characterizes how strongly the unit vectors drawn according to *f*_*h*_(**x** | *μ*, *κ*) are concentrated about the mean direction *μ*. In this work, we used the procedure for clustering directional data outlined in [22], and implemented in [24].

The principal ODF profile directions are extracted directly from the estimated clusters. The number of fibers in each voxel is automatically estimated from the reconstructed ODF profile by the vMF approach using the Bayesian Information Criterion (BIC) criterion [25]. In other words, “BIC” is used to decide on the number of components to select. All relevant statistical information about the ODF orientation and multiple fiber components may then be extracted from this fitting process.

## 3. Results and Discussion

### 3.1. Simulated Profiles

To validate our approach, we first simulated fiber crossing by generating diffusion images from the sum of two exponentials. For a given *b*-factor and noise level, we generate the diffusion-weighted signal *S*(**u**_*i*_) = ∑_*k*=1_^*n*^ *p*_*k*_ *e* ^−*b ***u**_*i*_^*T*^**D**_*k*_**u**_*i*_^ + noise, where **u**_*i*_ is the *i*th gradient direction on the sphere, *n* is the number of fibers, and **D**_*k*_ is the *k*th diffusion tensor profile rotated about the *z*-axis by a varying, user-specified angle. Each fiber was represented by a prolate diffusion tensor with typical eigenvalues {1700,200,200} (×10^−6^ mm^2^/s) [26], and *b*-values within the range *b* = {1500,6000} (s/mm^2^). We tested noise-free and noisy fiber profiles with Rician noise added to the simulated diffusion profile, for a typical standard deviation level of *σ* = 0.033, or signal-to-noise ratio SNR = *S*_0_/*σ* ≈ 30 with *S*_0_ = 1 [6]. Rician noise data was synthesized by *S* = ||*S*_*f*_ + *N*(0, *σ*), *N*(0, *σ*)||, where *S*_*f*_ is the synthesized noise-free signal, *N*(0, *σ*) is a random sample drawn from the normal distribution with mean 0 and standard deviation *σ*, and ||·|| is the *L*_2_-norm operator. This procedure is commonly used in other neuroscience toolkits [15, 16].

**Figure 1:**
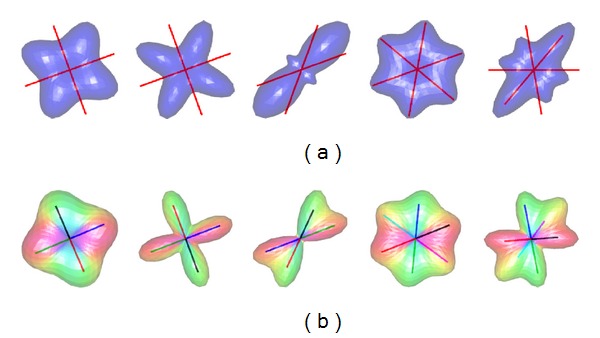
(a) Examples of synthesized noise free diffusion profiles. The red lines indicate the fiber directions used in the simulations. (b) Reconstructed fiber profiles from the simulated profiles in (a). The colored lines indicate the estimated fiber directions using mixtures of vMF distributions. The *b*-values used in the simulations were *b* = {1500,3000,5000,3000,5000}, and the angles between crossing fibers were [90°, 90°, 50°, 60°, 50°] (from left to right).

**Figure 2:**
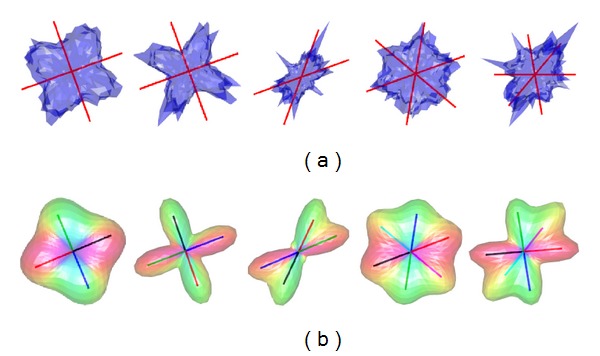
(a) Examples of synthesized noisy diffusion profiles, with Rician noise level *σ* = 0.033 (SNR ≈ 30). The red lines indicate the fiber directions used in the simulations. (b) Reconstructed fiber profiles from the simulated diffusion profiles in (a). The colored lines indicate the estimated fiber directions using mixtures of vMF distributions. The *b*-values used in the simulations were *b* = {1500,3000,5000,3000,5000}, and the angles between crossing fibers were [90°, 90°, 50°, 60°, 50°] (from left to right).

Sampling densities of *N* = 81 and *N* = 321 on the *S* ^2^ hemisphere, corresponding to a third and seventh-order tessellation of the icosahedron, were used in ODF profile mapping.  Figure 1(a) illustrates examples of synthesized noise-free diffusion profiles using the procedure described above with *N* = 321 on the *S* ^2^ hemisphere. These diffusion profiles were then used to reconstruct ODF profiles using the GQI method for reconstruction, and vMF mixtures for directional mapping, originating the estimated ODF profiles and fiber directions shown in Figure 1(b).  Figure 2 shows similar examples for noisy diffusion profiles synthesized with Rician noise level *σ* = 0.033. Profiles for two and three crossing fibers are illustrated in these figures. The angles for the three examples of two-fiber simulations shown in Figure 1(a) were {90°, 90°, 50°}, and the corresponding *b*-values were *b* = {1500,3000,5000}. The influence of the *b*-values in the reconstructed ODF profiles is clearly visible. For the two examples of three-fiber simulations illustrated in Figure 1(a), the angles between simulated fibers were 60° in one case, and 50° in the other case, with *b*-values *b* = 3000 and *b* = 5000, respectively. As shown, even with noisy profiles, crossing angles of 50°, and more than two crossing fibers, the clustered vMF procedure is able to correctly identify the underlying fiber orientations in the diffusion profiles.

### 3.2. Comparative Estimation Errors

To evaluate the precision and robustness of the procedures for estimating crossing fiber angles and selecting the correct number of fibers, we compared the proposed clustered vMF approach with a currently often used approach for crossing fiber mapping. As referred to in the Introduction, local maxima of the reconstructed ODF are often identified simply by selecting a large number of randomly sampled points on the sphere and searching within a fixed radius neighborhood [6]. For crossing fibers, the major fiber is identified by the largest local maximum (the global maximum), and the minor fiber is identified by the second largest local maximum [9]. We refer to this procedure as the standard (Std) approach. This Std procedure is implemented in several neuroimaging software packages. In this work, we have followed the implementation of the Std procedure used in the “DSI Studio” package, publicly available from the NITRC repository (http://www.nitrc.org/) (see also http://sites.google.com/a/labsolver.org/dsi-studio/).

**Figure 3:**
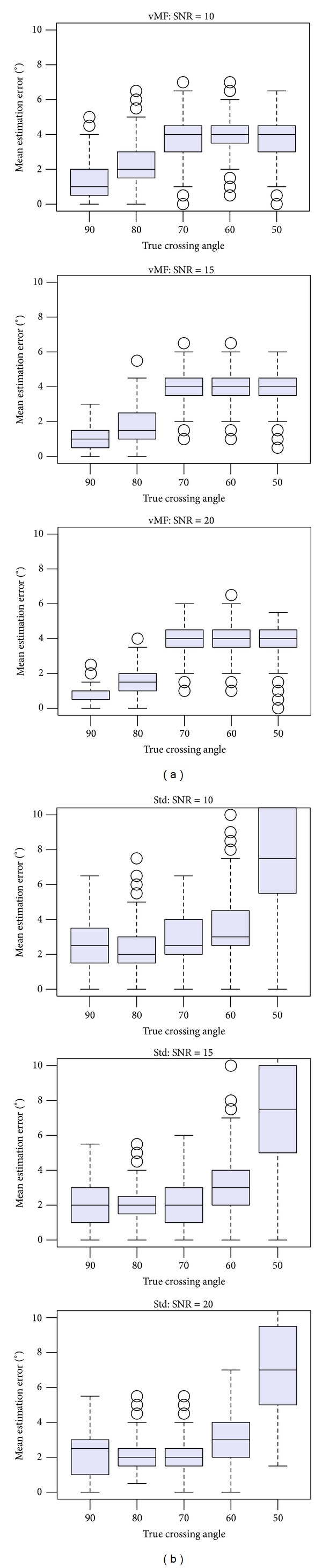
Boxplots representing the mean estimation error for various crossing angles between fibers and SNRs. Fiber compartments with weights *w*_*i*_ = {0.5,0.5},  *i* = 1,2, *b*-value = 3000, and threshold = 0.4 were used in the simulations. (a) refer to the clustered vMF method. (b) refer to the Std method.

**Figure 4:**
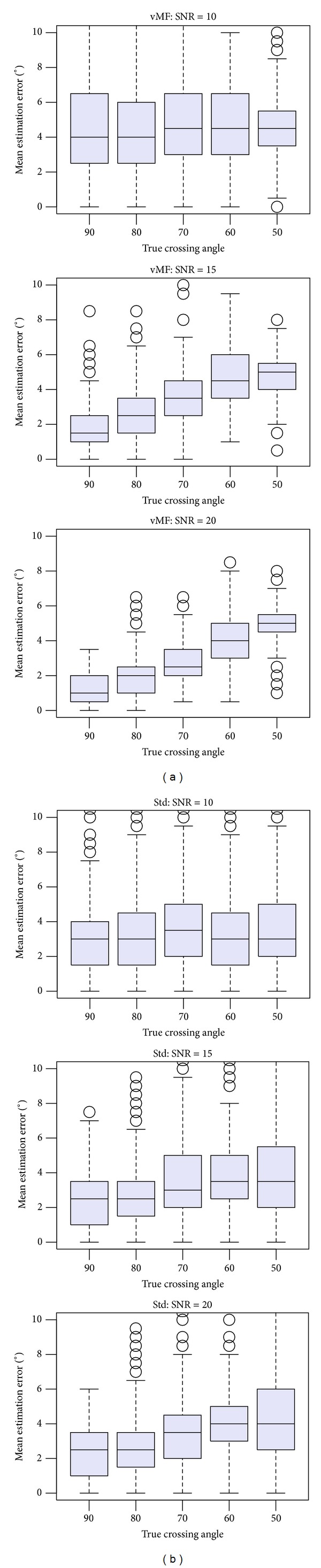
Boxplots representing the mean estimation error for various crossing angles between fibers and SNRs. Fiber compartments with weights *w*_*i*_ = {0.7,0.3},  *i* = 1,2, *b*-value = 6000, and threshold = 0.4 were used in the simulations. (a) refer to the clustered vMF method. (b) refer to the Std method.

We conducted statistical random tests for estimating fiber angles between crossing fibers. The tests simulated diffusion profile voxels with two-crossing fibers of different angles and SNRs typically encountered in diffusion MRI acquisitions, randomly positioned within the unit sphere. Each test used 600 samples for crossing angle estimation and selection of the number of fibers. For the clustered vMF approach, the automatic BIC selection procedure for estimating the number of fibers per voxel mentioned in Section 2.2 was applied. The boxplots on Figure 3(a) depict the angular precision and dispersion of the estimated fiber directions in degrees, for crossing angles between fibers of {90°, 80°, 70°, 60°, 50°}, and SNR = {10,20,30}, using the proposed clustered vMF approach. The boxplots on Figure 3(b) show equivalent results for the Std method. We performed the tests using sampling densities of *N* = 321 on the *S* ^2^ hemisphere, *b*-value = 3000, and threshold = 0.4. Angular precision is quantified by the average of the angular errors between the estimated fiber directions and the true ones inside the voxel. The results in Figure 3 were obtained with two fiber compartments with equal volume fractions, *w*_*i*_ = {0.5,0.5},  *i* = 1,2. To account for different volume fractions within the voxels, Figure 4 shows the results of the tests for fiber compartments with volume fractions *w*_*i*_ = {0.7,0.3},  *i* = 1,2. The tests reported in Figure 4 were conducted with SNR = {10,15,20}, and *b*-value = 6000.

Our results suggest that “clustered vMF” is globally more robust than the Std approach. The crossing angles are estimated with low dispersion for the range of crossing angles considered. In our tests, the Std method showed lower accuracy and precision than the clustered vMF method for crossing angles below 60° and low *b*-values. In these cases, the Std method is typically enhanced with better regularization procedures, higher *b*-values, or better ODF reconstruction approaches [27], to improve ODF peak resolution discrimination. The clustered vMF estimation shows low noise-sensitivity, stressing the advantage of statistical procedures over traditional deterministic procedures for directional fiber estimation.

### 3.3. Curved Fiber Bundle Simulation

**Figure 5:**
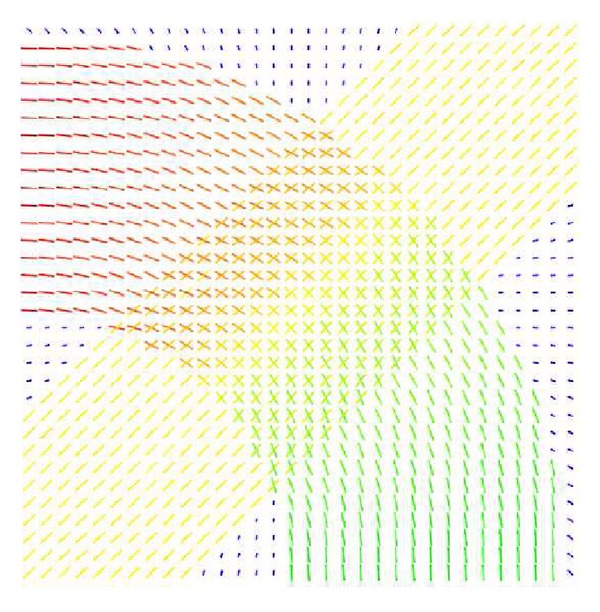
Reconstructed ODF orientations for the curved fiber bundle simulation with Rician noise level *σ* = 0.033 (SNR ≈ 30).

In [28] an example of two crossing fiber bundles was used to generate a synthetic tensor diffusion-weighted MRI field of dimension 32 × 32. In this example one of the fiber bundles is curved, to enable simulation of fiber bundles crossing for various intersection angles at each voxel. We have used a similar example to illustrate the reconstruction of fiber bundle orientations, using the GQI reconstruction and the vMF mixture procedures outlined in this paper with a *b*-value *b* = 4000.  Figure 5 illustrates the reconstructed ODF orientations for the curved fiber bundle simulation. This figure is the result of a Rician noise simulation with *σ* = 0.033 (SNR ≈ 30), showing that the crossing orientations at each simulated voxel have been correctly identified, paving the way to enhanced tractographic procedures.

### 3.4. Real Data Experiments

In this section we report on experiments using a DICOM data set provided by the “Advanced Biomedical MRI Lab, National Taiwan University Hospital.” Specifically, we have used the data set “DSI 203-point 2 mm” publicly available from http://dsi-studio.labsolver.org/download-images. This data set is from a normal 24-year-old male volunteer and has been provided as a demonstration data set in connection with the “DSI Studio” software for diffusion MR images analysis [9]. The data set was obtained with an echo planar imaging diffusion sequence with twice-refocused echo, dimension 96 × 96 × 60, and slice thickness 1.9 mm. Further details on the data set specification are available from the internet address mentioned above. We have tested our model with the two *b*-tables that accompanies the data set. One is a *b*-table for a *𝕊* ^2^-like grid denoted by “dsi203_bmax4000.txt.” The other is the *b*-table for the 3D-DSI sampling scheme used in the DICOM data acquisition. This *b*-table has 203 points uniformly distributed on a 3D grid limited to the volume of the unit sphere. In both tables, the *b*-values range from 0 to 4000. The ODF reconstructions were performed with 321 points uniformly distributed on the unit *𝕊* ^2^ hemisphere.

**Figure 6:**
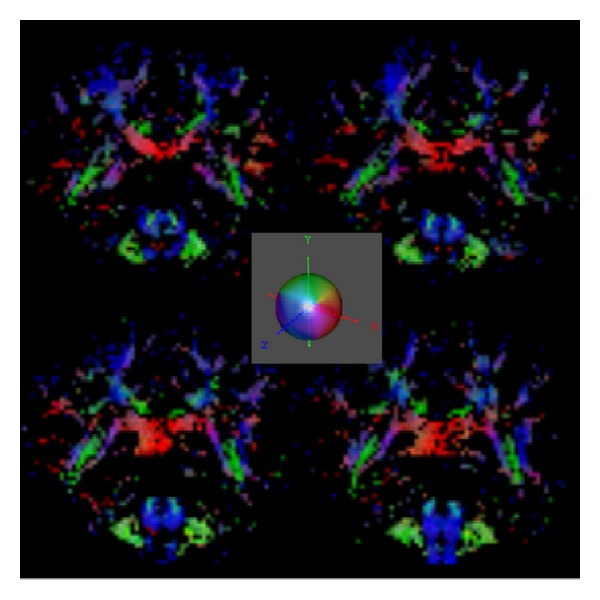
RGB maps (DEC-maps) for coronal slices {59,60}  (upper panels), and {61,62}  (lower panels), using the clustered vMF method for directional estimation and the GQI method for ODF reconstruction. The central inset panel illustrates directionally color encoding.

Using the GQI method outlined in Section 2.1, we obtained estimates of the ODFs at each voxel. The results reported in this section in connection with the application of the GQI method were obtained using the 3D-DSI sampling scheme for computing the GQI basis functions. These basis functions were then used to estimate the voxel's ODF profiles on *𝕊* ^2^ grids. To summarize anisotropic properties of the ODF and infer the underlying crossing patterns of the fibers we used the generalized fractional anisotropy (GFA) metric [9, 29]. A GFA threshold of 0.4 applied on the normalized ODF was used prior to visualization.  Figure 6 shows RGB (red-green-blue) color-maps to highlight directional information for coronal slices in range {59–62}, computed from the ODF principal directions using the clustered vMF procedure. This figure uses a GFA-modulated directionally encoded color (DEC) map [30] to highlight major fiber areas. The color encoding is illustrated in the central inset panel in Figure 6.

**Figure 7:**
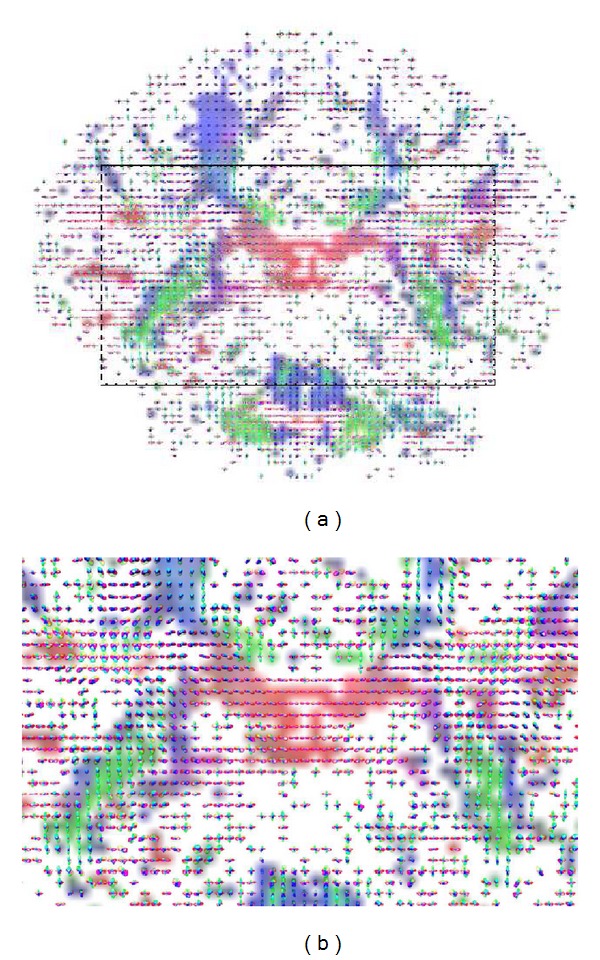
Glyph-map fields of estimated ODF profiles for voxels in coronal slice 60 (a). Zoomed image of the selected region marked by the black rectangle on (a), encompassing the ROIs depicted in Figure 8  (b).

**Figure 8:**
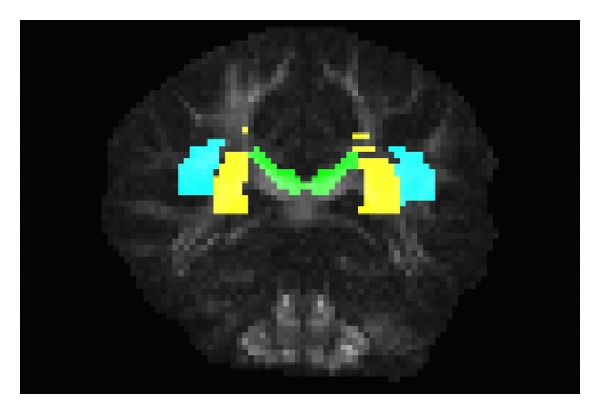
Coronal brain slice 60 showing the location of selected brain regions: CR—yellow areas, SLF—blue areas, and CC—green area, according to the “ICBM DTI-81 Atlas.”

Figure 7 shows glyph-map fields of estimated ODF profiles, for voxels in coronal slice 60, using sampling densities of *N* = 321 on the *S* ^2^ hemisphere. The black rectangle in Figure 7(a) delineates a region which includes the superior longitudinal fasciculus (SLF), superior corona radiata (CR), and the intersection with the left part of the body of the corpus callosum (CC).  Figure 7(b) depicts a zoomed image of the selected region. The localization of the selected brain regions within the slice is illustrated in Figure 8: CR—yellow areas, SLF—blue areas, and CC—green area. These areas were extracted with reference to the “ICBM DTI-81 Atlas,” LONI Laboratory of Neuro Imaging, UCLA, http://www.loni.ucla.edu/Atlases. These areas are typically fiber crossing regions, where major fiber tracts intersect at approximately orthogonal angles [31, 32]. The central greenish region distinctly maps the body of the corpus callosum, where the horizontal single-fiber orientation pattern is clearly identified.

**Figure 9:**
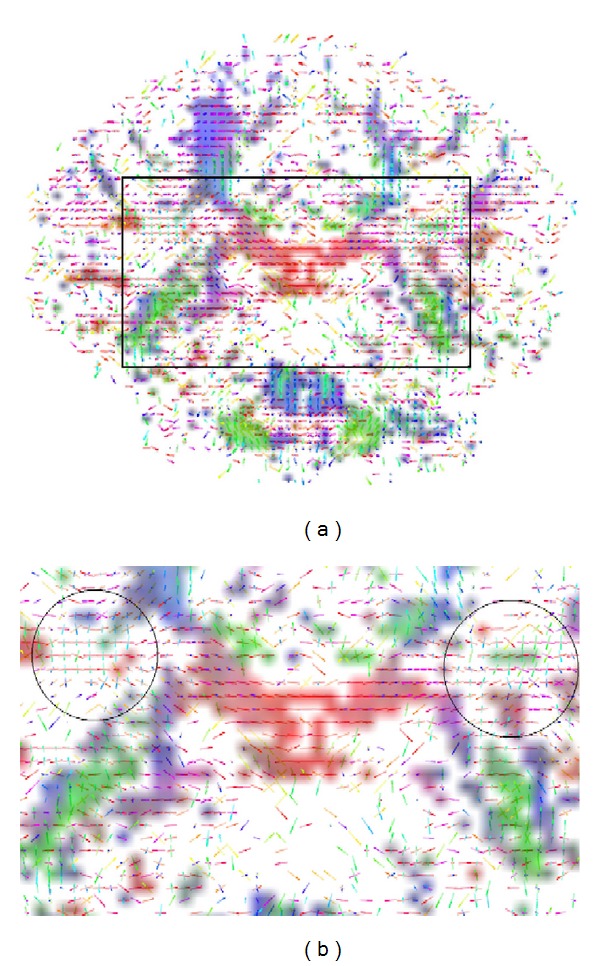
Line-maps for the field of profiles estimated by the clustered vMF method with GFA threshold equal to 0.4, for voxels in coronal slice 60, using a RGB map overlay (a). Zoomed image of the selected region marked by the black rectangle on (a), encompassing the ROIs depicted in Figure 8  (b). Circled areas mark voxel fields with high density of crossing fibers.

**Figure 10:**
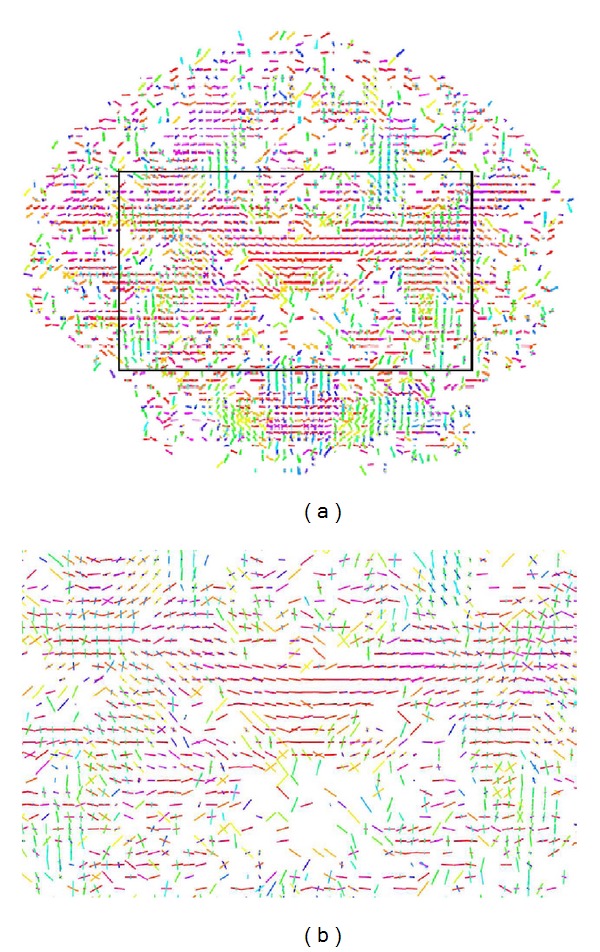
Line-maps for the field of profiles estimated by the clustered vMF method, for voxels in coronal slice 60, using a GFA map overlay (a). Zoomed image of the selected region marked by the black rectangle on (a), encompassing the ROIs depicted in Figure 8  (b).

**Figure 11:**
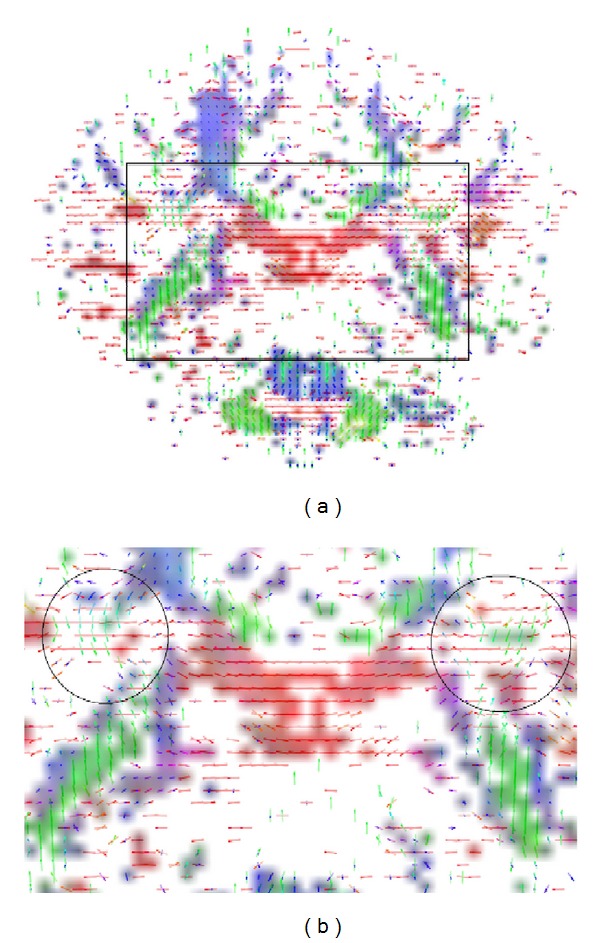
Line-maps for the field of profiles estimated by the Std method, for voxels in coronal slice 60, using a RGB map overlay (a). Zoomed image of the selected region marked by the black rectangle on (a), encompassing the ROIs depicted in Figure 8  (b). Circled areas mark voxel fields with high density of crossing fibers.

**Figure 12:**
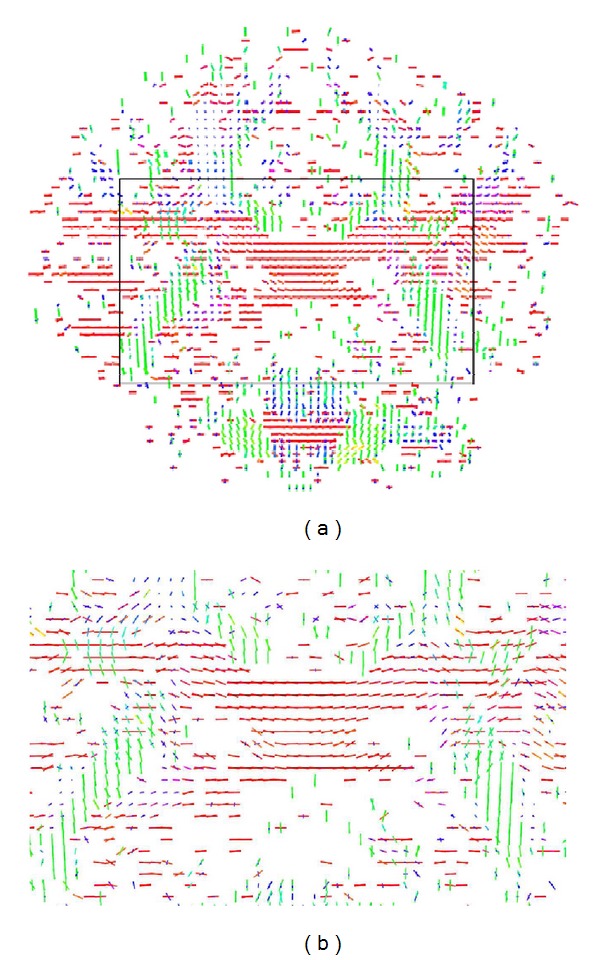
Line-maps for the field of profiles estimated by the Std method with GFA threshold equal to 0.4, for voxels in coronal slice 60 (a). Zoomed image of the selected region marked by the black rectangle on (a), encompassing the ROIs depicted in Figure 8  (b).

Figure 9(a) illustrates the voxel fiber orientations which were estimated for coronal slice 60, using the clustered vMF procedure outlined in Section 2.2 with GFA threshold equal to 0.4. In this figure the colored areas represent the RGB-map overlay to facilitate the location of important tissue structures. A zoomed image of the area marked by the black rectangle on Figure 9(a) is depicted in Figure 9(b). This area was selected to encompass the regions of interest (ROIs) depicted in Figure 8. For clarity of orientation visualization, Figure 10 visualizes the fields of line-mappings without overlays estimated by the clustered vMF method for coronal slice 60. For comparative purposes, similar figures using the Std method for orientation mapping are shown in Figures 11 and 12.

Figure 7(b) shows that the estimated orientation of the crossing fibers is consistent with the fiber tracts in the surrounding white matter tissue. This analysis is reinforced in the line-maps shown in Figures 9 and 10 for coronal slice 60. The circles depicted in Figures 9 and 11 mark voxel fields with high density of crossing fibers, based on the vMF and Std methods, respectively. Although a quantitative assessment of the best representation is difficult to evaluate with real data sets, it can be qualitatively observed that the clustered vMF method estimates a higher density of crossing fibers in these circled areas than the Std method.

**Figure 13:**
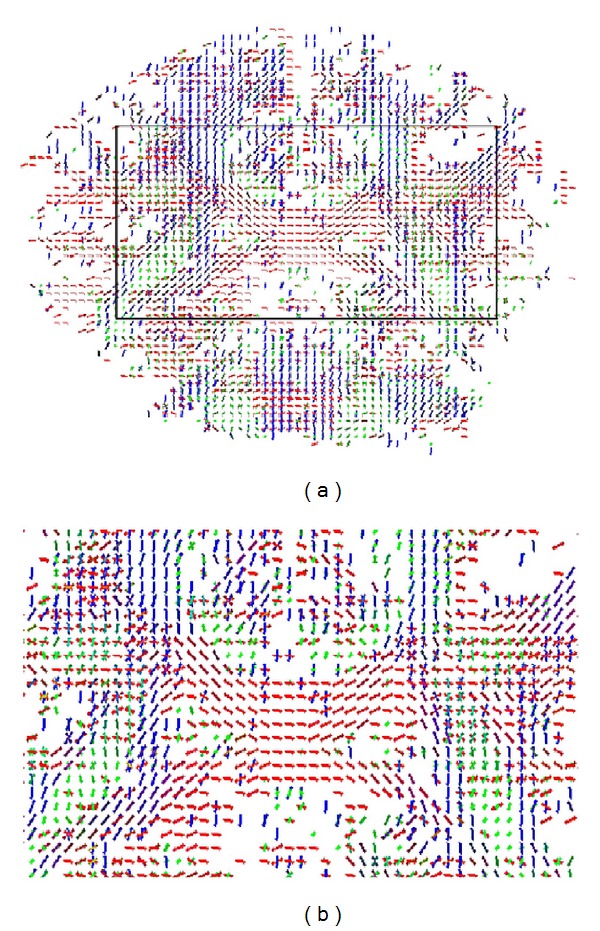
Line-maps for the field of profiles estimated with the “DSI Studio” package. The GQI method implemented in this package was applied to the same data, gradient table, and coronal slice as in Figures 9–12, using a QA threshold equal to 0.024 (a). Zoomed image of the selected region marked by the black rectangle on (a), encompassing the ROIs depicted in Figure 8  (b).

A comparison with the results obtained with the application of the “DSI Studio” package is illustrated in Figure 13. The GQI method implemented in “DSI Studio” was applied to the same data, gradient table, and coronal slice referenced above, yielding the results shown in Figure 13. “DSI Studio” uses the quantitative anisotropy (QA) measure [9], instead of the GFA measure, as fiber threshold parameter. The visualization shown in Figure 13 was drawn using a QA threshold equal to 0.024. Although the representations shown in Figure 13 and Figures 9–12 use different threshold indices, the line mappings appear qualitatively similar.

### 3.5. Implementation and Reproducible Research

The analyses and figures described in this work were performed using software programmed entirely in **R** [33]. The **R**-package **gdimap** [20] implements the reconstruction and clustered vMF estimation methodology described in this work and is freely available from the CRAN repository (http://CRAN.R-project.org/). The **R** language programming system has been the platform of choice for many researchers working in the neuroscience and neuroimaging fields [34]. **R** provides a reproducible research environment to many well-developed statistical tools needed for the analysis of neuroimaging data. In particular, the packages “oro.nifti” [35], “movMF” [24], and “rgl” [36], have been used for manipulating and visualizing medical imaging data. The package “oro.nifti” is used for reading and writing NIfTI formatted data sets; “movMF” provides support for fitting and simulating mixtures of von Mises-Fisher distributions; “rgl” is an OpenGL rendering device interface, which provides an interactive viewpoint navigation facility for the **R** programming language.

## 4. Conclusions

The approach of using the standard deterministic method, Std, for mapping fiber directions is pervasive in currently available packages. However, using deterministic procedures to support probabilistic fiber mapping jeopardizes rigorous fiber tractography and may originate deficient maps of white matter fiber networks. We should bear in mind that these packages are used regularly by hundreds of neuroimaging researchers, medical practitioners, and neuropsychologists, with scarce knowledge of the internal code workings and assumptions. We have proposed a new probabilistic method for estimating and mapping fiber directions from ODF reconstructions for the purpose of supporting probabilistic tractographic algorithms. In this approach, each estimated voxel fiber direction is associated with a component of the fitted mixture of vMF distributions. Each voxel fiber principal direction is defined by the summary statistics of the estimated vMF component in the mixture. Fully probabilistic tractography may now profit from this statistical information for fiber tracking. We perceive this approach as a major departure from current practices in fiber tracking methodologies.

Based on experiments, we have shown that by applying directional data clustering procedures to mixtures of vMF distributions multiple fiber configurations can be estimated in a robust manner, without recourse to ad hoc regularization procedures. The simulation tests show that this clustered vMF approach achieves good angular resolution, being able to discriminate multiple crossing fibers with relatively small separation angles (50°) and typical Rician noise levels. Using real data sets we found by inspection that the clustered vMF method estimates a higher density of intravoxel fiber crossings than the Std method in brain regions with multiple crossing fiber bundles. A denser net of crossing fibers in these regions is supported by histological studies. On the other hand, our tests suggest that the traditional method of extracting fiber directions based on local maxima of the reconstructed ODF suffers from both low accuracy and low precision for small crossing angles and/or low *b*-values. In these cases, appropriate regularization procedures or enhanced ODF reconstruction techniques should be carefully selected, before intravoxel fiber mapping is performed in order to mitigate these effects. However, regularization procedures are based on heuristics that are difficult to be appropriately tuned to the data at hand.

More comprehensive tests have to be conducted to evaluate the performance of the proposed clustered vMF method in other fiber configurations, such as fiber splitting or fiber kissing. In any case, the clustered vMF procedure is not restricted by considerations of voxel fiber symmetry, or odd number of fibers per voxel. The procedure applies statistical information criteria to decide on the number of components (voxel fibers) to select. The automatic selection criterion used for discriminating between single- and crossing-fiber voxel configurations, as well as for estimating the number of fibers per voxel, was found adequate in the tests. In summary, we think that statistical procedures tend be more robust than deterministic ones for diffusion MRI data, which is subject to different types of unspecified noise and partial volume averaging effects.

One of the main purposes of caring for rigorous fiber orientation mapping is to support the difficult task of tracing the trajectory of white matter fiber tracts *in vivo* [19]. We intend to build on the quantitative and qualitative information provided by the proposed directional statistics approach to support the study of white matter bundle networks in the human brain. In particular, this information may be combined with atlas-based methods to build robust tractographic algorithms for complex fiber configurations.
